# NMD‐insensitive *CDH1* mutation as a mechanism for retained E‐cadherin protein expression in lobular carcinoma in situ (LCIS)

**DOI:** 10.1111/his.15553

**Published:** 2025-09-05

**Authors:** Matthias Christgen, Stephan Bartels, Hans Kreipe

**Affiliations:** ^1^ Institute of Pathology Hannover Medical School Hannover Germany

**Abbreviations**
CDH1: gene symbol for E‐cadherin
ECH‐6: clone name for an anti‐;E‐cadherin antibody
IHC: immunohistochemistry
ILC: invasive lobular carcinoma
LCIS: lobular carcinoma in situ
LN: lobular neoplasia
mRNA: messenger RNA
NGS: next generation sequencing
NMD: nonsense‐mediated decay
PTC: premature termination codon

Dear Editors,

The inactivation of the *CDH1* tumour suppressor gene through somatic mutation, leading to subsequent loss of E‐cadherin protein expression, is the key oncogenic driver in lobular carcinoma in situ (LCIS) and invasive lobular carcinoma (ILC) of the breast. A vigorous debate exists regarding the role of E‐cadherin immunohistochemistry (IHC) in diagnosing LCIS and ILC. Some experts advocate for the routine use of E‐cadherin IHC, supporting broad indications for ancillary immunohistochemical staining, as it improves inter‐pathologist agreement.^1^, ^2^ Others, however, caution against its overuse, citing concerns about oversimplified binary interpretations of IHC results and challenging cases where morphology and E‐cadherin status appear discordant.^3^ To contribute to a better understanding of such challenging cases, we present an instructive example of an E‐cadherin‐positive LCIS harbouring a distinctive *CDH1* alteration that might be of broad interest.

A 44‐year‐old female underwent diagnostic vacuum‐assisted breast biopsy. Histomorphology examination revealed an intra‐acinar proliferation of non‐cohesive epithelial cells filling and expanding nearly all acini of at least 20 scattered lobules (Figure 1A). The morphology was consistent with lobular neoplasia (LN), specifically LCIS. However, IHC using the anti‐E‐cadherin antibody clone ECH‐6 demonstrated membranous E‐cadherin expression rather than the expected E‐cadherin loss (Figure 1B). E‐cadherin staining intensity was minimally weaker than in the background normal breast epithelium. Suspecting an unusual *CDH1* alteration, we performed next‐generation sequencing (NGS) on DNA extracted from micro‐dissected LCIS foci. This revealed a pathogenic nonsense‐mediated decay (NMD)‐insensitive *CDH1* mutation introducing a premature termination codon (PTC) in the last exon of the *CDH1*/E‐cadherin gene (p.D834*) (Figure 2A). NMD has received little attention in the context of E‐cadherin loss so far. NMD is an mRNA surveillance mechanism in eukaryotic cells. NMD degrades aberrant transcripts carrying PTCs.^4^ Most somatic *CDH1* mutations, whether nonsense or frameshift, generate PTCs that trigger NMD. When combined with loss of heterozygosity (LOH), this mechanism leads to the E‐cadherin‐negative phenotype typically observed in LCIS and ILC. However, NMD is inefficient for PTCs located beyond a critical boundary—50 nucleotides upstream of the last intron–exon junction.^4^ This biological principle applies universally to all human genes, including *CDH1*. In *CDH1*, mutations with PTCs downstream of codon 800 fall into the NMD‐insensitive region (Figure 2A).^5^ NMD‐insensitive mutations are translated into truncated E‐cadherin proteins lacking the cytoplasmic tail but retaining epitopes detected by anti‐E‐cadherin antibodies such as clone ECH‐6 (which binds between amino acid positions 594–697).^5^ This explains the retained, membranous E‐cadherin expression observed in our case. While NGS provides valuable insights, it is not essential for correctly identifying and classifying such cases in routine diagnostics. The absence of the beta‐catenin binding site (located at amino acid positions 811–882) in truncated E‐cadherin proteins leads to the characteristic loss of beta‐catenin expression that typically accompanies *CDH1* inactivation (Figure 1C).^2^ IHC for p120‐catenin can be challenging to interpret, often displaying a partially membranous or partially cytoplasmic staining pattern (Figure 1C). We have previously reported a similar NMD‐insensitive *CDH1* mutation (p.E841*) in an E‐cadherin‐positive ILC case^6^. Although such NMD‐insensitive *CDH1* mutations are notable for their E‐cadherin‐positive immunophenotype, they are relatively rare. In an in silico analysis of *CDH1*‐mutated LCIS and ILC cases from our laboratory at Hannover Medical School and the cBioPortal database, NMD‐insensitive mutations with PTCs beyond codon 800 accounted for only 10 out of 275 cases (3.6%) (Figure 2B).

**Figure 1:**
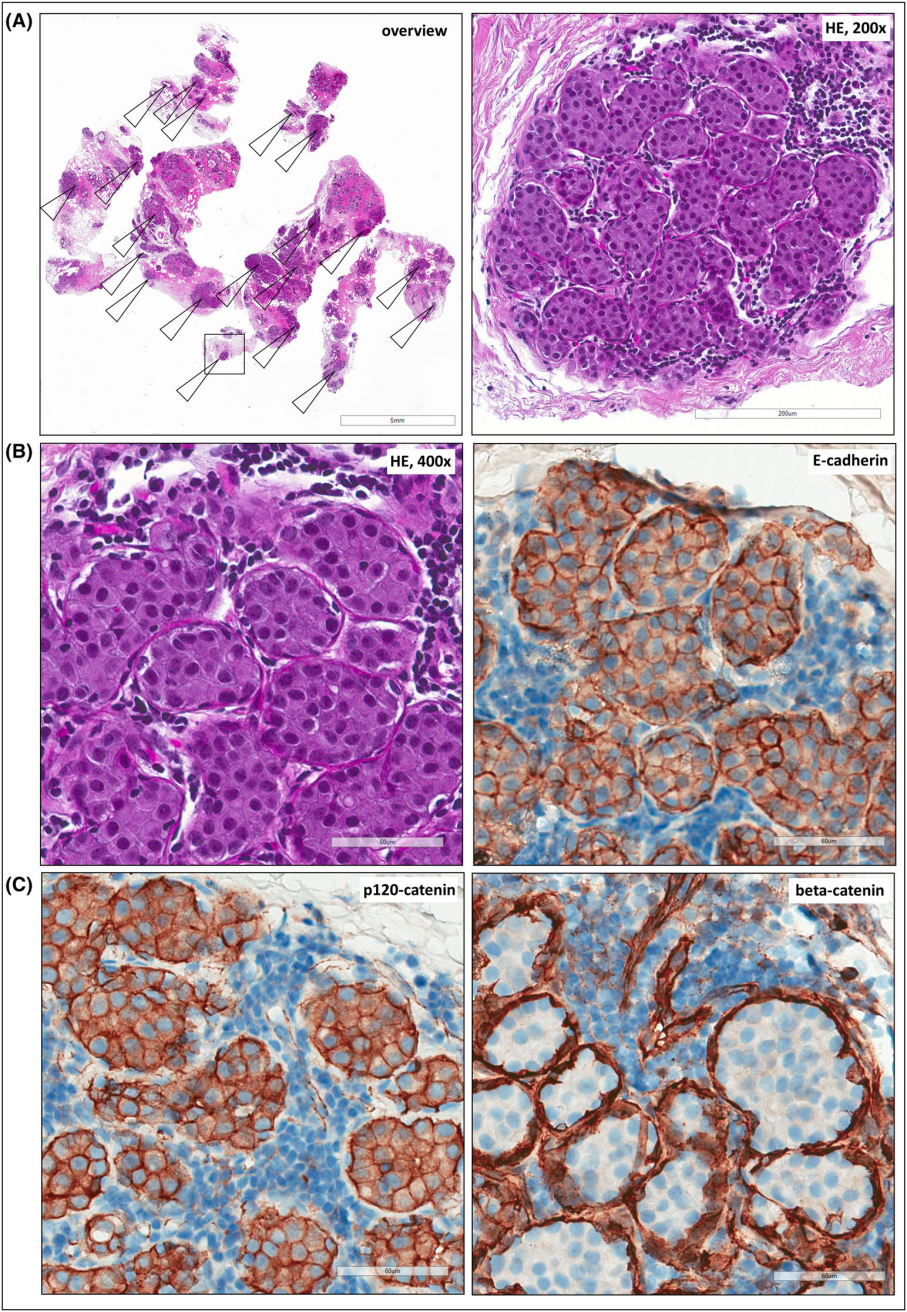
(**A**) Histology of a diagnostic vacuum‐assisted breast biopsy. Shown is an overview of the HE‐stained section (left) and details at ×200 magnification (right). Arrows indicate at least 20 lobules affected by LCIS. The scale bar corresponds to 5 mm (left) or 200 μm (right). Informed consent was obtained from the corresponding patient. (**B**) HE‐stained section (left) and immunohistochemistry for E‐cadherin (right, antibody clone ECH‐6) at ×400 magnification. The scale bar corresponds to 60 μm. (**C**) Immunohistochemistry for p120‐catenin (antibody clone 98) and beta‐catenin (antibody clone 14) at ×400 magnification. The scale bars correspond to 60 μm. Please note that p120‐catenin shows a membranous staining pattern combined with a suspicious cytoplasmic hue.

**Figure 2:**
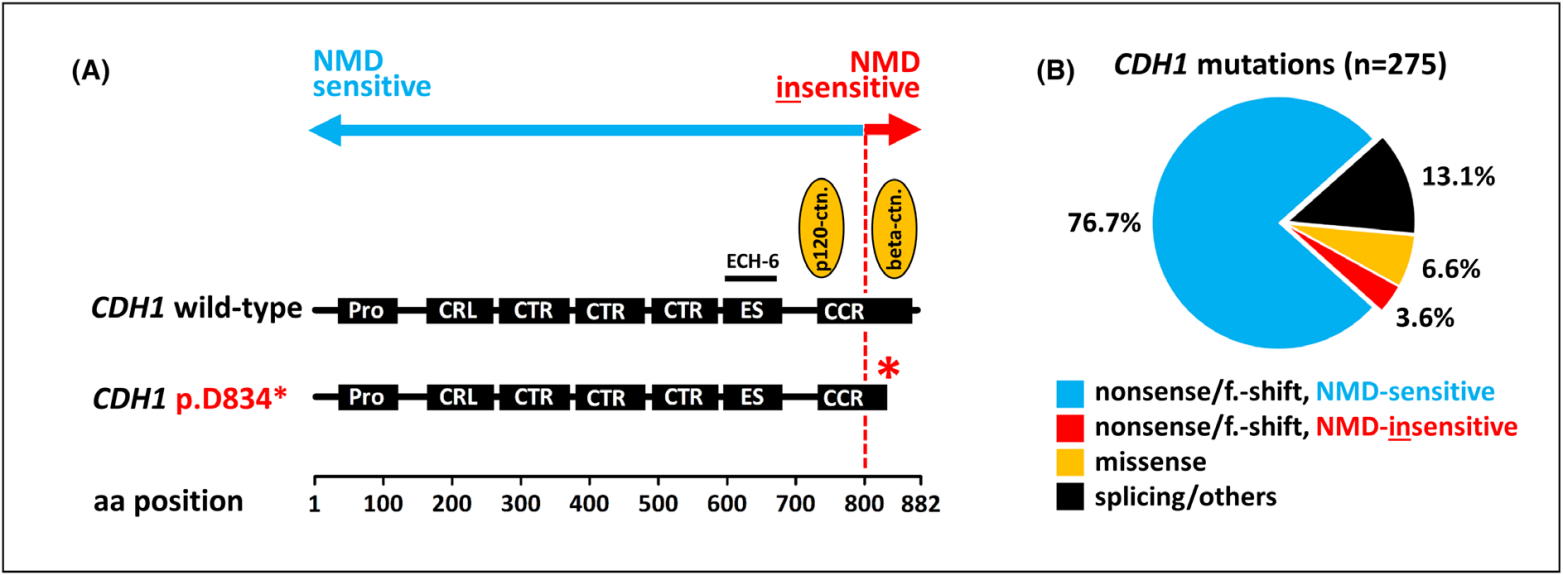
(**A**) Next‐generation sequencing results. Shown is the structure of the E‐cadherin protein. The red asterisk highlights that the *CDH1* p.D834* mutation introduces a PTC that truncates the cytoplasmic tail of the E‐cadherin protein and abrogates the beta‐catenin binding site. E‐cadherin domains are as follows: Pro, prodomain; CRL, cadherin repeat‐like domain; CTR, cadherin tandem repeat domain; ES, early set domain; CCR, cadherin cytoplasmic region. The red dotted line highlights the NMD boundary located approximately at codon 800. (**B**) In silico analysis. The frequency of NMD‐insensitive *CDH1* mutations with PTCs beyond codon 800 (NMD boundary) was determined in a compilation of *n* = 275 *CDH1*‐mutated LCIS or ILC cases registered at the Hannover Medical School (*n* = 134) or at the cBioPortal platform (*n* = 141) (https://www.cbioportal.org/). aa, amino acid; f.‐shift., frameshift.

In conclusion, while LCIS and ILC are typically E‐cadherin‐negative, rare exceptions exist. One such mechanism—NMD‐insensitive *CDH1* mutation—can result in preserved E‐cadherin expression, as illustrated in our case. This underscores the importance of integrating morphology and IHC findings for accurate diagnosis. For classic LCIS, histologic work‐up should include a thorough morphologic evaluation and IHC for E‐cadherin. In extraordinary LCIS cases with seemingly discordant features between morphology and immunophenotype, ancillary immunohistochemical staining for beta‐catenin and molecular analyses can provide an additional, objective confirmation. This principle aligns with recent consensus recommendations on the use of IHC in breast pathology.^1^

## Author contributions

MC and HK performed the morphologic evaluation. SB performed the genetic analysis. All authors contributed to the writing of the manuscript.

## Funding statement

This work was performed without funding.

## Conflict of interests

The authors declare no conflict of interest.

## Data Availability

Data are available upon reasonable request.
